# Emergence and rapid global dissemination of CTX-M-15-associated *Klebsiella pneumoniae* strain ST307

**DOI:** 10.1093/jac/dky492

**Published:** 2018-12-04

**Authors:** Kelly L Wyres, Jane Hawkey, Marit A K Hetland, Aasmund Fostervold, Ryan R Wick, Louise M Judd, Mohammad Hamidian, Benjamin P Howden, Iren H Löhr, Kathryn E Holt

**Affiliations:** 1 Department of Biochemistry and Molecular Biology, Bio21 Molecular Science and Biotechnology Institute, University of Melbourne, Parkville, VIC, Australia; 2 Department of Medical Microbiology, Stavanger University Hospital, Stavanger, Norway; 3 Department of Clinical Science, University of Bergen, Bergen, Norway; 4 The ithree Institute, University of Technology Sydney, Ultimo, NSW, Australia; 5 Microbiological Diagnostic Unit Public Health Laboratory, Department of Microbiology and Immunology, University of Melbourne at The Peter Doherty Institute for Infection and Immunity, Parkville, VIC, Australia; 6 London School of Hygiene and Tropical Medicine, London, UK

## Abstract

**Objectives:**

Recent reports indicate the emergence of a new carbapenemase-producing *Klebsiella pneumoniae* clone, ST307. We sought to better understand the global epidemiology and evolution of this clone and evaluate its association with antimicrobial resistance (AMR) genes.

**Methods:**

We collated information from the literature and public databases and performed a comparative analysis of 95 ST307 genomes (including 37 that were newly sequenced).

**Results:**

We show that ST307 emerged in the mid-1990s (nearly 20 years prior to its first report), is already globally distributed and is intimately associated with a conserved plasmid harbouring the *bla*_CTX-M-15_ ESBL gene and several other AMR determinants.

**Conclusions:**

Our findings support the need for enhanced surveillance of this widespread ESBL clone in which carbapenem resistance has occasionally emerged.

## Introduction

Several reports have indicated the recent emergence of a new MDR *Klebsiella pneumoniae* (*Kp*) clone, ST307. We have recently generated data suggesting that ST307 is becoming an important cause of ESBL-producing *Kp* infections in Norway (M. A. K. Hetland, A. Fostervold, I. H. Löhr on behalf of The Norwegian Study Group on *Klebsiella pneumoniae*, unpublished data), and others have reported it as an emerging cause of KPC-producing *Kp* infections.^1–3^

Here we summarize what is known in the literature and investigate 95 geographically diverse ST307 whole-genome sequences from 11 countries to better understand the emergence and global molecular epidemiology of this clone and identify the antimicrobial resistance (AMR) genes with which it is associated.

## Materials and methods

### Ethics

The proposed collection of novel clinical *Kp* isolates from Norway was reviewed and approved by the Regional Committees for Medical and Health Research Ethics West (Norway, application ID: 2017/1185).

### Literature search

We searched PubMed for abstracts containing the words ‘ST307’ with/without ‘*Klebsiella pneumoniae*’ as of April 2018. ST307-isolate genomes were identified from our collections and public databases using Kleborate.^4^ In total, 549 published and 37 newly sequenced genomes were identified, representing 11 countries on five continents. However, we were unable to find corresponding literature reports for 45 of the 58 genome assemblies identified among those deposited in GenBank (as of December 2017) and hence these genomes were excluded from comparative analyses.

### Sequencing

Novel genomes were sequenced on the Illumina MiSeq platform generating 150 or 250 bp paired end (PE) reads as described previously.^5^ The oldest isolate (Kp616 from Iran, 2009) was also subjected to long-read Oxford Nanopore sequencing and hybrid genome assembly using Unicycler v0.4.4.^6^^,^^7^ The completed Kp616 genome comprised a 5246307 bp chromosome plus two plasmids (pKp616_1, 58 kbp; pKp616_2, 55 kbp; GenBank accession number GCA_003076555.1).

A core chromosomal single-nucleotide variant (SNV) alignment was generated using RedDog (reference: Kp616 chromosome) as described previously.^5^ Recombination was removed using Gubbins.^8^ A preliminary tree made using FastTree^9^ indicated that the majority of strains from Texas (*n *=* *451/468, 96.4%) formed a distinct monophyletic clade; hence, we randomly selected one isolate per year to represent this clade in comparative analyses. The final recombination-free alignment of 1465 SNVs in 95 genomes was subjected to Bayesian phylogenetic analysis using BEAST 2 v2.4.7^10^ as described previously.^5^ A GTR, relaxed clock, constant population size model was determined to be the best fit and we confirmed a strong temporal signal by date randomization and linear regression (Figure S1, available as Supplementary data at *JAC* Online).

*De novo* genome assemblies were generated with Unicycler v0.4.4.^6^ AMR genes and virulence loci were detected using Kleborate.^4^^,^^11^^,^^12^ Capsule synthesis (K) and lipopolysaccharide (O) loci were typed using Kaptive.^13^

Sample information accession numbers, citations and genotyping results for the 95 genomes included in the final analyses are listed in Table S1. Notably, this collection represents the most diverse sample of ST307 *Kp* genomes to date, including isolates (i) from diverse sources, e.g. human infections [urine (*n *=* *34), blood (*n *=* *31), respiratory (*n *=* *10) and unknown/other (*n *=* *17)], two human rectal carriage isolates and one isolate from canal water; and (ii) from diverse geographies, including 47 genomes (49.5%) from seven countries not represented in previous comparative analyses.^1^^,^^2^

## Results and discussion

**Figure 1.**
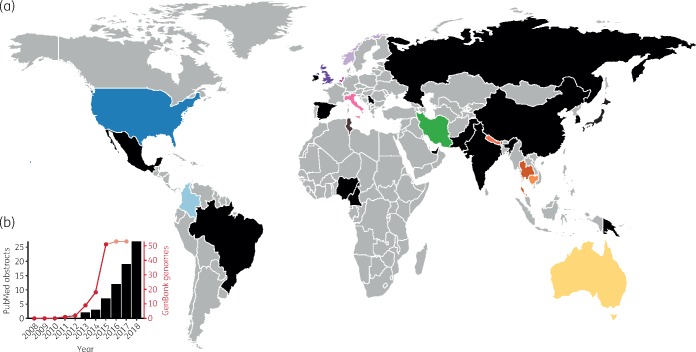
Geographical distribution and increasing reports of ST307. (a) Countries of collection of ST307 isolates reported in the literature, the international *K. pneumoniae* MLST database and/or for which genome data are available (also see Table S2). Countries for which isolate genomes were included in the current analysis are coloured as shown in Figure 2. All other countries where ST307 has been reported are coloured black. (b) Reports of ST307 in the literature and among genome assemblies deposited in GenBank. Black bars show the cumulative number of PubMed abstracts as of April 2018, identified using the search criteria ‘ST307’ with/without ‘*Klebsiella pneumoniae*’ (Table S2). The red line shows the cumulative number of isolates for which genome assemblies are deposited in GenBank as of December 2017. Dates indicate year of isolate collection, not date of deposition, hence recent values will likely increase as further genomes are deposited. The first ST307 isolate reported in the international MLST database was collected in 2008.

A total of 26 literature reports were identified by systematic search and a further 6 papers were identified by association with published genome sequences (Table S2). The oldest recorded ST307 isolate was collected in The Netherlands in 2008 (*Kp* MLST database; https://bigsdb.pasteur.fr/klebsiella/klebsiella.html) but the earliest clinical strain reported in the literature was collected in Pakistan in 2009.^14^ This was followed by sporadic isolations across Europe, Asia, Africa and the Americas, the majority (>98%) from a variety of human clinical specimens plus a minority from other sources, e.g. human rectal samples, companion animals and environmental/sewage water samples (summarized in Table S2 and Figure 1). Several reports indicated local dissemination of ST307 harbouring KPC genes, *bla*_KPC-2_ (Columbia, USA, South Korea) and *bla*_KPC-3_ (Italy)^3^^,^^15–18^ while an analysis of >1700 ESBL-producing *Kp* from a hospital network in Texas, USA found high prevalence of *bla*_CTX-M-15_-positive ST307 strains, ∼1/3 of which also carried *bla*_KPC-2_ genes and three carried *bla*_KPC-3_.^1^ This was consistent with other reports that *bla*_CTX-M-15_ is common in ST307^1–3^^,^^14^^,^^19^ (also see Table S2). Aside from *bla*_KPC_ genes, carbapenem resistance conferred by NDM-1 or OXA-48 carbapenemases has been reported,^1–3^^,^^19^^,^^20^ as has resistance to the novel β-lactam inhibitor combination ceftazidime/avibactam^21^ and colistin.^20^^,^^22^

We performed the first molecular dating analysis for ST307 using the 95 genomes in our final subsampled collection. This indicated that ST307 emerged in 1994 [95% highest posterior density (HPD), 1974–2006], close to the emergence date estimated previously for ST258,^23^ despite the fact that the latter was reported in the literature and recognized as a disseminated clone more than a decade earlier than ST307.^24^ The estimated mutation rate for ST307 (1.18 × 10^−6^ substitutions/site/year, 95% HPD, 8.01 × 10^−^^7^–1.58 × 10^−6^; Figure S1) was remarkably similar to that estimated previously for ST258 (1.03 × 10^−6^ substitutions/site/year, 95% HPD 8.09 × 10^−^^7^–1.24 × 10^−6^)^23^ but faster than that of hypervirulent *Kp* ST23 (3.40 × 10^−7^ substitutions/site/year, 95% HPD 2.43 × 10^−^^7^–4.38 × 10^−7^),^5^ which represent the only *Kp* clones for which comparable analyses have been published to date.

**Figure 2.**
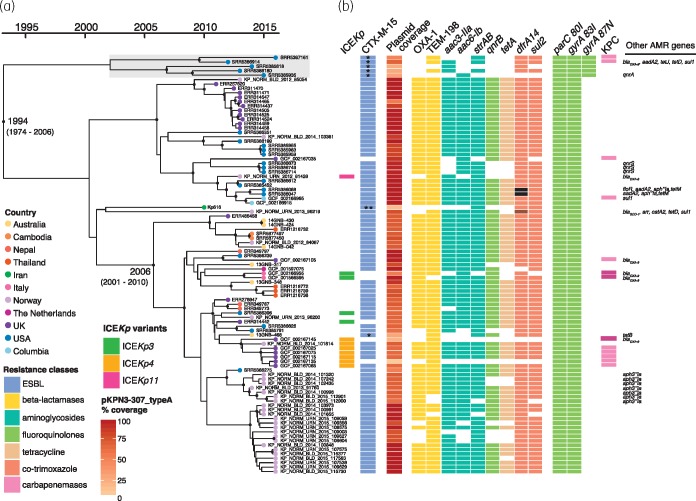
Bayesian phylogeny of 95 ST307 isolates. (a) Dated phylogeny of ST307 isolates (*n *=* *95), with tips coloured by country of isolation (as shown in the inset legend and Figure 1). Black dots on internal nodes indicate ≥95% posterior probability. Light grey shading shows subsampled Texan-specific clade. (b) Presence of the yersiniabactin-carrying ICE*Kp* elements (variants coloured as shown in the inset legend), antimicrobial resistance genes (blocks coloured by drug class), and coverage of *bla*_CTX-M-15_ plasmid pKPN3-307_typeA. In the CTX-M-15 column: * indicates *bla*_CTX-M-15_ is inserted in chromosome; ** *bla*_CTX-M-15_ inserted in IncN plasmid. Brown in the *dfrA14* column indicates *dfrA27* allele and black the *dfrA12* allele. In the KPC column, light pink indicates *bla*_KPC-2_ and dark pink indicates *bla*_KPC-3_. These data are available for interactive viewing at https://microreact.org/project/ryiY_FlfQ.

The phylogeny revealed two deep-branching lineages, one of which has become globally distributed, comprising genomes from the Americas, Asia, Australia, the Middle East and Europe (including the 12 genomes reported by Villa *et al*.^2^; see Figure 2 and also available for interactive viewing at https://microreact.org/project/ryiY_FlfQ). Within this lineage there was evidence of transfer of ST307 between countries, and for all countries with three or more genomes there were multiple clusters within the global lineage. The countries with the highest representation were distributed most broadly (Norway, *n *=* *30; USA, *n *=* *22; UK, *n *=* *22), suggesting that the same patterns would likely be detected for most countries if sampling was increased (Figure 2). The second lineage included only subsampled strains from Texas (2011–15, shaded grey in Figure 2), indicating that the majority of the hundreds of infections attributed to ST307 in the report by Long *et al*.^1^ resulted from prolonged local transmission of this clade, a finding that was not previously evident because the genomes were not compared with those from other geographies. Texan isolates were also found in the global lineage, suggesting that the USA may be a potential origin for ST307, as most of its genetic diversity was present in that location.

We used Kleborate^4^ to detect *Kp* virulence determinants that are positively associated with invasive infections.^11^^,^^12^^,^^25^ While these determinants are most commonly detected among drug-susceptible ‘hypervirulent’ *Kp*, they are occasionally found among MDR strains,^11^^,^^12^ posing a risk of severe, difficult-to-treat infections. We detected no evidence of the *Kp* virulence plasmid (encoding the salmochelin and aerobactin siderophores plus RmpA/RmpA2),^26^ but a minority of genomes (*n *=* *12, 12.6%) harboured the yersiniabactin siderophore locus located within three distinct chromosomally integrated ICE*Kp* variants (ICE*Kp3*, ICE*Kp4* and ICE*Kp11*). Hence our data indicate a lower prevalence of ICE*Kp* than that previously reported for ST307 (66%, 8 of 12 genomes investigated^2^). The distribution of ICE*Kp* insertions on the ST307 core genome tree indicated more than four independent acquisitions with limited expansion of recipient sub-lineages (Figure 2), consistent with the patterns recently reported for ST258 and other common MDR *Kp* clones.^11^ Unlike these other clones,^13^^,^^23^ all ST307 shared the same K and O loci (KL102, associated with *wzi* allele 173, and O2v2), both of which have also been identified among various other *Kp* STs, including those from other disseminated MDR clones, e.g. ST105, ST152 and ST1583.^12^^,^^13^ An additional putative capsule synthesis locus (Kp616 genes C2861_20465 to C2861_20520^2^), which was previously reported in ST307^2^ and is unlike any known K locus, was also conserved (present at ≥95% nucleotide identity and ≥90% coverage in 95/95 genomes) but is rare among the broader *Kp* population (just 10 *Kp* nucleotide blast matches were identified in the GenBank nucleotide database).

In contrast to the virulence loci, acquired AMR genes were highly prevalent; 93 (97.9%) isolates carried acquired resistance determinants associated with three or more drug classes (Table S1). The ParC 80I and GyrA 83I fluoroquinolone resistance-associated mutations were conserved in all genomes. The *bla*_CTX-M-15_ ESBL gene was found in 89 (93.7%) genomes, and 81 (85.3%) harboured it in combination with *sul2*, *dfrA14* and *strAB* with/without *aac(3)-IIa*, which were all linked to an MDR plasmid (see below). *bla*_KPCs_ and other AMR genes were occasionally identified (Figure 2 and Table S1). For the majority of genomes carrying *bla*_CTX-M-15_ (*n *=* *88/89), BLASTn confirmed that this gene was located downstream of IS*Ecp1*, which forms a transposon to mobilize *bla*_CTX-M-15_ and promotes its expression.^27^ Four complete ST307 IncFII_K_/IncFIB_K_*bla*_CTX-M-15_ plasmids have been published,^2^ and share an insertion of the IS*Ecp1*/*bla*_CTX-M-15_ transposon within Tn*3*. Read-mapping to the largest of these plasmids, pKPN3-307_typeA (accession KY271404) and assembly graph inspections of our *bla*_CTX-M-15_-positive genomes showed that all carried the same IS*Ecp1*/*bla*_CTX-M-15_ transposon. In 82/89 cases, the same pKPN3-307_typeA IncFII_K_/IncFIB_K_ plasmid backbone was present and IS*Ecp1*/*bla*_CTX-M-15_ was located in the same site within Tn*3*, consistent with conservation of the same IncFII_K_/IncFIB_K_ ESBL plasmid (including various deletion variants; Figure S2). The exceptions were as follows: (i) an Australian isolate (13GNB-468) had the IS*Ecp1*/*bla*_CTX-M-15_ transposon inserted in the chromosomal gene *feoB*; (ii) Kp616 carried no pKPN3-307_typeA-like plasmid but harboured *bla*_CTX-M-15_ on an IncN plasmid; and (iii) the five representatives of the Texas-specific lineage carried two chromosomal insertions of the IS*Ecp1*/*bla*_CTX-M-15_ transposon (within Kp616 loci C2861_02545 and C2861_22795, not detailed in the previous study^1^). This, coupled with the additional GyrA 87N fluoroquinolone resistance mutation in the Texan lineage, may have contributed to its prolonged transmission in the hospital setting by facilitating enhanced resistance to antimicrobials without any burden of plasmid maintenance. Regardless of these exceptions, the level of plasmid conservation in ST307 is remarkable, mirroring the association of ST258 with the *bla*_KPC_ pKpQIL plasmid^23^ and suggesting that the plasmid confers limited fitness cost to the host (although plasmid-positive, *bla*_CTX-M-15_-negative genomes were observed; Table S1).

Complementing the increasing reports in the literature, our analyses reveal that ST307 is a highly successful MDR clone that shares many traits with ST258 (i.e. date of emergence, evolutionary rate, high prevalence of AMR genes, low prevalence of ICE*Kp*s but multiple independent acquisitions, and high conservation of a single plasmid), but is closely associated with *bla*_CTX-M-15_ rather than *bla*_KPCs_. With sufficient exposure ST307 can acquire and disseminate carbapenemases,^1–3^ and likely other clinically important AMR determinants. Our analyses show for the first time that ST307 is readily transferred between countries and has already become globally disseminated but remained largely unnoticed for almost 20 years. These findings indicate an urgent need for enhanced surveillance of MDR *Kp* to monitor ST307 alongside other well-known clones and detect emerging MDR threats.

## Supplementary Material

Supplementary Data

Supplementary Table 1
