# Supplementary material for: Emergence and rapid global dissemination of CTX-M-15-associated Klebsiella pneumoniae strain ST307
Source: J Antimicrob Chemother. 2018 Dec 4;74(3):577–81. doi: 10.1093/jac/dky492 (PMC6376852; doi:10.1093/jac/dky492)
Supplement: Supplementary Data [file dky492_supplementary_data.doc]

**Supplementary data**

**Emergence and rapid global dissemination of CTX-M-15-associated *Klebsiella pneumoniae* strain ST307**

Kelly L. Wyres^1a*^, Jane Hawkey^1a*^, Marit A. K. Hetland^2^, Aasmund Fostervold^2,3^, Ryan R. Wick^1^, Louise M. Judd^1^ , Mohammad Hamidian^4^, Benjamin P. Howden^5^, Iren H. Löhr^2b^, Kathryn E. Holt^1,6b^

^1^ Department of Biochemistry and Molecular Biology, Bio21 Molecular Science and Biotechnology Institute, University of Melbourne, Parkville, VIC, Australia

^2^ Department of Medical Microbiology, Stavanger University Hospital, Stavanger, Norway

^3^ Department of Clinical Science, University of Bergen, Bergen, Norway

^4^ The ithree Institute, University of Technology Sydney, Ultimo, NSW, Australia

^5^ Microbiological Diagnostic Unit Public Health Laboratory, Department of Microbiology and Immunology, University of Melbourne at The Peter Doherty Institute for Infection and Immunity, Parkville, VIC, Australia

^6^ London School of Hygiene and Tropical Medicine, London, UK

a/^b^ These authors contributed equally to this work.

*corresponding author

Corresponding author details:

kwyres@unimelb.edu.au

Department of Biochemistry and Molecular Biology, Bio21 Molecular Science and Biotechnology Institute, University of Melbourne, Parkville, Australia

***Contents***

Table S2: Reports of ST307 *K. pneumoniae*; A) from the literature; B) among genome assemblies deposited in Genbank and not yet described in the literature.

Figure S1: Temporal signal amongst ST307 genomes.

Figure S2: Bayesian phylogeny of ST307 isolates and plasmid content.

Please see separate Excel file for Table S1.

**Table S2:** **Reports of ST307 *K. pneumoniae*; A) from the literature; B) among genome assemblies deposited in Genbank and not yet described in the literature**

| PMID / Accession(s) | Year published/  deposited | Year of collection | Country | # Isolates  (# Genomes*) | Carbapenemases | ESBLs | Source (host) |
| --- | --- | --- | --- | --- | --- | --- | --- |
| A |  |  |  |  |  |  |  |
| **23530541** | 2013 | 2010 | USA | 2 | KPC-2 | - | Hospital; blood culture (human) |
| **23622882** | 2013 | 2009-2010 | Pakistan | 12 | - | CTX-M-1 phylogroup | Hospital; urine, wound (human) |
| **23722448**** | 2013 | 2011 | UK | 1 | - | CTX-M-15 | NS |
| **24476498** | 2014 | 2013 | Italy | 2 | KPC-3 | - | Hospital; rectal swab (human) |
| **26341707** | 2015 | 2011 | Tunisia | 2 | - | CTX-M-15 | Hospital; urine (human) |
| **25712531**** | 2015 | 2012 | Nepal | 3 (3) | - | CTX-M-15 | Hospital; respiratory, tissue, catheter tip (human) |
| **26207318** | 2015 | 2013 | Korea | 14 | - | CTX-M-15 | Hospital; urine (human) |
| **26177547** | 2015 | 2014 | Italy | 27 | KPC-3 | CTX-M-15 | Hospital (human) |
| **26503660** | 2015 | 2013-2014 | Colombia | 17 | KPC-2, KPC-3 | CTX-M-15, TEM-11 | Hospital; bloodstream, intra-abdominal, respiratory, surgical site, skin and soft tissue, UTI, other (human) |
| **26782324** | 2016 | 2009 | Brazil | 1 | - | CTX-M-15 | Sewage water from wastewater treatment plant (environmental) |
| **26996563** | 2016 | 2012 | South Korea | 4 | - | CTX-M-15 | Hospital (human) |
| **26518051** | 2016 | 2013 | Spain | 1 | OXA-48 | CTX-M-15 | LTCFs; rectal swab (human) |
| **27056222** | 2016 | 2014 | Italy | 1 (1) | KPC-3 | CTX-M-15 | Hospital; urine (human) |
| **26864946**** | 2016 | 2014 | The Netherlands | 1 (1) | - | CTX-M-15 | Hospital; blood (human) |
| **27446056** | 2016 | 2015 | Japan | 3 | - | CTX-M-15 | Veterinary hospital; pus, urine (dog, cat) |
| **28210248** | 2017 | 2015 | China | 1 | NDM-1(NE), OXA-48 | CMY-6, CTX-M-15 | Hospital; wound of a burn patient (human) |
| **29176971** | 2017 | 2015 | Italy | 1 | KPC-2 | - | Well (environmental) |
| **28242665** | 2017 | 2015 | Serbia | 1 | OXA-48 | CTX-M-15 | Hospital; tracheal aspirate (human) |
| **28185686** | 2017 | 2015 | South Korea | 42 | KPC-2 | CTX-M-14, CTX-M-15 | Hospital; blood, body fluid, respiratory, pus, rectal swab, urine (human) |
| **28893788** | 2017 | 2016 | Colombia | 1 | - | CTX-M-11 | Hospital; blood (human) |
| **28223459**** | 2017 | 2011 | UK | 1 (1) | - | CTX-M-15 | Hospital; blood (human) |
| **28512093**** | 2017 | 2011-2015 | USA | 526 (468) | KPC-2, KPC-3, NDM-1, OXA-23 | CTX-M-15, CTX-M-27, SHV-12, TEM-15, TEM-143, TEM-164, TEM-168 | Hospital; blood, respiratory, urine, other (human) |
| **28636666** | 2017 | 2014-2015 | Mexico | 1 | NDM-1 | - | Hospital; outbreak (human) |
| **28118859**** | 2017 | 2015 | Thailand | 4 (4) | - | CTX-M-15 | Hospital; blood (human). Environment (canal) |
| **28785421** | 2017 | 2014-2016 | United Kingdom | 8 | KPC-2, KPC-3 | CTX-M-15 | Hospital (human) |
| **29471690** | 2018 | 2012 | United Arab Emirates | 1 | NDM-1, OXA-162 | CTX-M-15 | Hospital; urine (human) |
| **29479341** | 2018 | 2015 | Guinea | 1 | - | CTX-M-15 | Rectal swab (rats) |
| **29479347** | 2018 | 2016 | Cameroon | 1 | - | SHV-134 | Hand swab of abattoir worker (human) |
| **29651277***** | 2018 | 2010-2015 | South Korea | 7 | NDM | - | Hospital; body fluids, blood, pulmonary, rectal swabs, urine, wounds, other (human) |
| **29373087** | 2018 | 2010-2015 | Tunisia | 3 | NDM-1 | CTX-M-15 | Hospital; urine, catheter (human) |
| **29571838** | 2018 | 2014-2015 | Italy | 7 | - | CTX-M-15 | LTCFs; urine, diarrheic faecal (human) |
| **29422888** | 2018 | 2014-2015 | South Korea | 167 | KPC-2, KPC-4 | - | Hospital (human) |
| **29478336** | 2018 | 2014-2015 | South Korea | 97 | KPC-2, KPC-4, NDM-1 | - | Hospital; body fluid, blood, pus, respiratory, urine (human) |
| **29263067** | 2018 | NS | USA | 7 | KPC-2 | CTX-M-15 | Hospital (human) |
| B |  |  |  |  |  |  |  |
| **GCF_000567685.1 GCF_000821465.1 GCF_000821605.1 GCF_000822005.1 GCF_000823125.1** | 2014 | NS | NS | 5 | - | CTX-M-15 | NS |
| **GCF_001903615.3 GCF_001902865.3** | 2016 | 2013 | USA | 2 | KPC2 | CTX-M-15 | Urine (Human) |
| **GCF_900085725.1** | 2017 | 2011 | NS | 1 | - | CTX-M-15 | Blood (human) |
| **GCF_002283495.1** | 2017 | 2018 | Columbia | 1 |  | CTX-M-15 | Blood (human) |
| **GCF_002235215.1 GCF_002235175.1 GCF_002235035.1** | 2017 | 2013-2014 | Italy | 3 | KPC-2, KPC-3 | CTX-M-15 | Blood, urine, respiratory (human) |
| **GCF_002188055.1 GCF_002187305.1** | 2017 | 2014 | Nigeria | 2 | - | CTX-M-15 | Blood (human) |
| **GCF_002239875.1** | 2017 | 2016 | Thailand | 1 | - | CTX-M-63 | Urine (human) |
| **GCF_900172545.1 GCF_900172585.1 GCF_900172615.1 GCF_900172645.1 GCF_900172655.1 GCF_900172685.1 GCF_900172705.1 GCF_900172735.1 GCF_900172775.1 GCF_900172785.1 GCF_900172815.1 GCF_900172875.1 GCF_900172885.1 GCF_900172905.1 GCF_900172925.1 GCF_900172975.1 GCF_900173075.1 GCF_900173105.1 GCF_900173125.1 GCF_900173135.1 GCF_900173375.1 GCF_900173415.1 GCF_900173945.1 GCF_900173955.1 GCF_900173965.1** | 2017 | 2015 | UK | 25 | - | CTX-M-15 | Blood, faeces (human), environment (NS) |
| **GCF_001903695.3 GCF_002185575.1 GCF_002186255.1 GCF_002184915.1 GCF_002186005.1** | 2017 | 2012-2014 | USA | 5 | KPC-2 | CTX-M-15 | Blood, urine (human) |

**a,** PubMED abstracts as of April 2018 identified using the search criteria “ST307” with/without “*Klebsiella pneumoniae*”. * Number of genomes reported in this work and included in our current analyses. ** Abstract did not meet search criteria but genomes described in the publication were identified as ST307 using *Kleborate* and were included in our comparative analyses; *** Abstract did not meet search criteria but was known to report ST307 *K. pneumoniae*. **b,** Genome assemblies desposited in GenBank as of Dec 2017 and identified as ST307 using *Kleborate.* These genomes were not included in our comparative analyses since we were not able to identify any associated publications in the literature. ESBL – Extended spectrum beta-lactamase; NS – Not stated; NE – Not expressed; LTCFs – Long term care facilities. Note that absence of a gene indicates that it was not present, or was not reported.

**Figure S1: Temporal signal amongst ST307 genomes. a,** Linear regression of year of isolation vs. root-to-tip distances from the maximum likelihood phylogeny for all 95 ST307 isolates in the final analysis. **b,** Mutation rate estimates and 95% HPD intervals for BEAST runs with real dates (red) and randomised dates (black).

**Figure S2: Bayesian phylogeny of ST307 isolates and plasmid content. a,** Dated phylogeny of 95 ST307 isolates reproduced from **Figure 2**, with tips coloured by country of isolation. Black dots on internal nodes indicate ≥95% posterior probability. **b,** Presence (black) or absence (white) in each genome of plasmid replicons from the PlasmidFinder database. **c,** Presence (grey) or absence (white) of genes in *bla*_CTX-M-15_-associated reference plasmid pKPN3-307_typeA (GenBank accession KY271404).
